# Living alone and antidepressant medication use: a prospective study in a working-age population

**DOI:** 10.1186/1471-2458-12-236

**Published:** 2012-03-23

**Authors:** Laura Pulkki-Råback, Mika Kivimäki, Kirsi Ahola, Kaisla Joutsenniemi, Marko Elovainio, Helena Rossi, Sampsa Puttonen, Seppo Koskinen, Erkki Isometsä, Jouko Lönnqvist, Marianna Virtanen

**Affiliations:** 1 Finnish Institute of Occupational Health, Topeliuksenkatu 41 a A, 00250 Helsinki, Finland; 2 Unit of Personality, Work and Health Psychology, Institute of Behavioral Sciences, University of Helsinki, P.O. Box 9, 00014 Helsinki, Finland; 3 Department of Epidemiology and Public Health, University College London, 1-9 Torrington Place, WC1E 6BT London, UK; 4 National Institute for Health and Welfare, P.O. Box 30, 00271 Helsinki, Finland; 5 Department of Psychiatry and Helsinki University Central Hospital, University of Helsinki, P.O. Box 20, 00014 Helsinki, Finland

**Keywords:** Mental health, Antidepressant medication, Living arrangement, Psychosocial factors, Socioeconomic

## Abstract

**Background:**

An increasing proportion of the population lives in one-person households. The authors examined whether living alone predicts the use of antidepressant medication and whether socioeconomic, psychosocial, or behavioral factors explain this association.

**Methods:**

The participants were a nationally representative sample of working-age Finns from the Health 2000 Study, totaling 1695 men and 1776 women with a mean age of 44.6 years. In the baseline survey in 2000, living arrangements (living alone vs. not) and potential explanatory factors, including psychosocial factors (social support, work climate, hostility), sociodemographic factors (occupational grade, education, income, unemployment, urbanicity, rental living, housing conditions), and health behaviors (smoking, alcohol use, physical activity, obesity), were measured. Antidepressant medication use was followed up from 2000 to 2008 through linkage to national prescription registers.

**Results:**

Participants living alone had a 1.81-fold (CI = 1.46-2.23) higher purchase rate of antidepressants during the follow-up period than those who did not live alone. Adjustment for sociodemographic factors attenuated this association by 21% (adjusted OR = 1.64, CI = 1.32-2.05). The corresponding attenuation was 12% after adjustment for psychosocial factors (adjusted OR = 1.71, CI = 1.38-2.11) and 9% after adjustment for health behaviors (adjusted OR = 1.74, CI = 1.41-2.14). Gender-stratified analyses showed that in women the greatest attenuation was related to sociodemographic factors and in men to psychosocial factors.

**Conclusions:**

These data suggest that people living alone may be at increased risk of developing mental health problems. The public health value is in recognizing that people who live alone are more likely to have material and psychosocial problems that may contribute to excess mental health problems in this population group.

## Background

The proportion of one-person households has doubled during the past three decades, with every third person in the U.S. and the U.K. living alone [1,2]. It has been estimated that by 2020, nearly 40% of all households will have only one inhabitant [3]. At the same time, there has been a dramatic increase in the consumption of antidepressant medications [4,5]. The potentially catastrophic consequences of single living were evident during the 1995 heat wave in Chicago, when most of the deaths occurred among elderly people living alone [6]. However, the long-term consequences of single living are poorly understood, because never before in history has there been such a great proportion of people living alone.

Living alone has been associated with psychological disadvantages and an increased risk of mental health problems, higher rates of consumption of psychotropic drugs [7-12], and a higher risk of suicide [13] compared to living with other persons in the same household. A prospective study demonstrated that household structure, that is, the social composition of people who reside in the same household, was a more important determinant of well-being than marital status in middle-aged people [14]. However, most studies in this field of research have been cross-sectional and have concentrated on selected populations such as elderly people living alone [15,16] or single parents [17]. Little is known about the mental health outcomes associated with single living in economically active people who represent the majority of the working-age population [13,18].

Several possible reasons may explain why people living alone suffer from poorer mental health, and why people living with others generally have better mental health. Living with other persons may offer emotional support, feelings of social integration, as well as tangible factors that protect against mental health problems [16,19,20]. Living alone, in turn, may be associated with psychosocial deficits such as feelings of isolation [13,19-22] and a lack of social integration and trust, which, in turn, are risk factors for mental health [23-25]. Single people may also face distress due to socioeconomic disadvantages, such as financial difficulties, and they may be prone to adverse health behaviors [19,21,22,26]. In working-age people, single living may be associated with poorer well-being in working life which, in turn, is known to be a predictor of mental health problems [24,27]. We are aware of no previous study that has examined aspects of working life as potential contributors to the association between living arrangements and mental health outcomes.

In the present study, we used antidepressant medication as a proxy measure for the most common mental disorders [28], as antidepressants are indicated for both depression and anxiety-related conditions. Our aim was two-fold: to examine 1) whether living alone was associated with antidepressant use during a 7-year period, and 2) the extent to which psychosocial, socioeconomic, and behavioral factors explained any observed association between living alone and the use of antidepressant medication. This study was based on data from Finnish working-age (30 to 65 years) men and women who formed a representative sample of the Finnish working population.

## Methods

### Participants

The participants were men and women from the Health 2000 Study [29], which was a two-stage stratified cluster sample representative of the Finnish mainland population aged 30 years or over. The base sample comprised 5871 participants between 30 and 65 years of age. We included those who were currently working or who had been working during the previous 12 months, thus reflecting current or recent economic activity. The sample with complete information on all study variables consisted of 3471 participants. Previously, it has been shown that the drop-outs had more depressive symptoms, were more often male, lived alone, and were more often economically inactive than those who participated [11,30]. The respondents received an information leaflet and gave their written informed consent. The Health 2000 Study was approved by the Ethics Committee for Epidemiology and Public Health in the Hospital District of Helsinki and Uusimaa in Finland, and the study was performed in accordance with the ethical standards of the Declaration of Helsinki.

### Measures

*Living arrangements *were examined at the baseline examination between August 15, 2000 and February 28, 2001. The participants were asked: "How many persons live in your household, including you?" The participants were classified as "living alone" if they reported a household size of one person, and "not living alone" if they reported a household size of two or more persons. The validity of this question was examined by cross-tabulating living arrangements with self-reported marital status. Of the 504 persons living alone, 184 were divorced, 25 widows, and 295 lived alone without a specific reason. There were 13 married persons living alone; we decided to retain them among the 504 persons who were classified as living alone, as they might have been separated but not yet divorced.

*Information on antidepressant medications *purchased at pharmacies between January 1, 2000 and December 31, 2008 was obtained from the National Prescription Register managed by the Social Insurance Institution of Finland. This register covers the entire outpatient population and all reimbursed doctor-prescribed medications in Finland. Participants who had purchased at least one prescription coded as N06A (the WHO Anatomical Therapeutic Chemical classification code for antidepressants) were considered as antidepressant users. Three variables were formed: baseline users (who had purchased at the study baseline between 2000 and 2001), users at follow-up (who had purchased between 2002 and 2008), and incident users (who had purchased between 2002 and 2008, but not at baseline).

*Psychosocial factors *included self-reports of a poor job climate (4-item questionnaire) [31], a lack of support at the workplace (2-item scale) [32], a lack of social support in private life (4-item scale) [33], and cynical hostility (8-item scale) [34]. Participants not currently working were asked to report the working conditions during the previous 12 months. For all psychosocial factors, the highest tertile represented those rated high in the characteristic. There were two main reasons for using categorical variables instead of continuous ones: (1) it enabled a better comparison with previous studies using similar measures [27,35], and (2) the assumption underlying the use of continuous variables is that each increment in the distribution equally adds risk, but this is not necessarily the case, as threshold effects are possible in a study where the outcome is a dichotomous mental health variable.

*Sociodemographic factors *were a low occupational grade (manual-level employee versus non-manual), a lack of educational qualifications (lack of secondary-level education versus having a degree), a low household income (as defined by the OECD: income per consumption unit below 50% of the national median), currently being outside work life (unemployed/home-maker/student/retired versus currently having a job), urban residency (living in a city or it's outskirts versus rural living), rental living (renting versus owning one's accommodation), and poor housing conditions (having ≥ 2 housing disadvantages including noise, draft, dirt, damp, chilliness, and fear versus having < 2 disadvantages).

*Health risk behaviors *included daily smoking (non-smokers versus smokers), heavy alcohol use (moderate users versus heavy users: > 20 g/day for women, > 40 g/day for men), low physical activity (any activity, including walking, ≥ 4 per week representing "high activity" versus < 4 times per week representing "low activity"), and obesity (body mass index < 30 versus ≥ 30).

### Data analysis

The data were analyzed using SAS 9.1 survey procedures and SUDAAN 9 software. This software applies weighting adjustments and sampling parameters to account for the clustering of a stratified sample. We used logistic regression analysis to examine the association of the living arrangement (alone vs. not alone) with the odds for having purchased reimbursed prescriptions of antidepressant medication. These analyses examined a) the cross-sectional use of antidepressants (baseline purchases of antidepressants as the outcome), b) the longitudinal use of antidepressants (purchases of antidepressant medication at any time during the 7-year follow-up period), and c) incident use of antidepressants (starting to use antidepressants during the 7-year follow-up period). We entered socioeconomic factors, psychosocial factors, and health behaviors as separate blocks in the age- and gender-adjusted models, and we calculated the percentage change in the odds ratio (OR) to evaluate their influence on the association between living arrangements and antidepressant use. These analyses were conducted in all participants, and additionally stratified by gender.

## Results

Characteristics of the study variables according to gender are presented in Table 1. Of the 3471 participants, 14.5% reported living alone, with an equal proportion of men and women living alone. The prevalence of antidepressant use in all participants was 6.2% at baseline, and altogether 17.2% used antidepressants at some point during the 7-year follow-up period. Each year, 1% to 2% of participants started to use antidepressants, and the prevalence of incident use during the entire follow-up period was 12.9% (year-by-year figures are provided in Additional file 1: Annex Table 1). Women had a greater prevalence and incidence of antidepressant use than men. Compared to women, men had less social support at work and a lower occupational grade than women, more often lived in rural areas, smoked more often, and used alcohol more heavily than women.

**Table 1:** Characteristics of the study variables

	n (%)			
	
	Men (n = 1695)	Women (n = 1776)	All (n = 3471)	*P *for gender difference
**Number of participants living alone**	244 (14.4)	260 (14.6)	504 (14.5)	0.838
**Number of antidepressant users:**				
At baseline	75 (4.4)	140 (7.9)	215 (6.2)	**< 0.001**
During 7-year follow-up	235 (13.9)	362 (20.4)	597 (17.2)	**< 0.001**
Incident users during 7-year follow-up	175 (10.3)	245 (13.8)	420 (12.1)	**0.002**
**Psychosocial factors:**^**a**^				
Poor job climate	622 (36.7)	604 (34.0)	1226 (35.3)	0.098
Lack of support at the workplace	618 (36.5)	551 (31.0)	1169 (33.7)	**0.001**
Lack of social support in private life	600 (35.4)	626 (35.2)	1226 (35.3)	0.926
High cynical hostility	455 (26.8)	466 (26.2)	921 (26.5)	0.687
**Sociodemographic factors:**				
Low occupational grade (blue-collar)	753 (44.4)	431 (24.3)	1184 (34.1)	**< 0.001**
Lack of secondary education	387 (22.8)	384 (21.6)	771 (22.2)	0.391
Low income (< 50% of national median)	182 (10.7)	213 (12.0)	395 (11.4)	0.244
Temporarily outside working life^b^	122 (7.2)	170 (9.6)	292 (8.4)	**0.012**
Urban residency	1058 (62.4)	1193 (67.2)	2251 (64.9)	**0.003**
Living at rent	379 (22.4)	402 (22.6)	781 (22.5)	0.846
Poor housing conditions^c^	152 (9.0)	185 (10.4)	337 (9.7)	0.149
**Health behaviors:**				
Daily smokers	490 (28.9)	380 (21.4)	870 (25.1)	**< 0.001**
Heavy alcohol use^d^	241 (14.2)	135 (7.6)	376 (10.8)	**< 0.001**
Infrequent physical activity^e^	336 (19.8)	424 (23.9)	760 (21.9)	**0.004**
Obesity (BMI ≥ 30)	310 (18.3)	340 (19.1)	650 (18.7)	0.519

The Health 2000 Study, n = 3471

^a^The highest tertile is the cut-off point

^b^Unemployed, home-maker, full-time student, or retired during past 12 months

^c^At least 2 of the following: draft, noise, dust or dirt, dampness, chilliness, crowding, fear

^d^According to WHO definintion: > 20 g for women per day, > 40 g for men per day

^e^Less than 4 times per week of any activity, including moderate activity such as walking

Table 2 presents psychosocial, demographic, and behavioral factors according to living arrangements. Participants living alone experienced a worse job climate, less support in private life, had a higher level of cynical hostility, were more often non-employed, lived in urban areas, were more likely to live in rental accommodation, had worse housing conditions, were more often smokers and used alcohol more heavily than those who did not live alone.

**Table 2:** Comparison of psychosocial, socioedmographic and behavioral factors in participants not living alone versus those living alone

	n (%)			
	
Characteristic	Not living alone	Living alone	All	*P *for alone
	(n = 2967)	(n = 504)	(n = 3471)	vs. not alone
**Psychosocial factors:**^**a**^				
Poor job climate	1016 (34.3)	210 (42.0)	1226 (35.3)	**0.002**
Lack of support at the workplace	999 (33.8)	170 (34.0)	1169 (33.7)	0.923
Lack of social support in private life	984 (33.3)	242 (48.2)	1226 (35.3)	**< 0.001**
High cynical hostility	760 (25.8)	161 (32.3)	921 (26.5)	**0.004**
**Sociodemographic factors:**				
Low occupational grade (blue-collar)	996 (34.1)	188 (38.1)	1184 (34.1)	0.089
Lack of secondary education	648 (22.2)	123 (24.7)	771 (22.2)	0.238
Low income (< 50% of national median)	338 (11.2)	57 (11.3)	395 (11.4)	0.940
Temporarily outside working life^b^	238 (7.8)	54 (10.5)	292 (8.4)	0.060
Urban residency	1873 (63.3)	378 (74.8)	2251 (64.9)	**< 0.001**
Living at rent	560 (18.7)	221 (43.4)	781 (22.5)	**< 0.001**
Poor housing conditions^c^	272 (9.1)	65 (12.7)	337 (9.7)	**0.029**
**Health behaviors:**				
Daily smokers	716 (24.3)	154 (30.7)	870 (25.1)	**0.002**
Heavy alcohol use^d^	302 (10.4)	74 (15.0)	376 (10.8)	**0.005**
Infrequent physical activity^e^	635 (21.3)	125 (24.6)	760 (21.9)	0.107
Obesity (BMI ≥ 30)	554 (18.9)	96 (18.9)	650 (18.7)	0.990

The Health 2000 Study, n = 3471

^a^The highest tertile is the cut-off point

^b^Unemployed, home-maker, full-time student, or retired during past 12 months

^c^At least 2 of the following: draft, noise, dust or dirt, dampness, chilliness, crowding, fear

^d^According to WHO definintion: > 20 g for women per day, > 40 g for men per day

^e^Less than 4 times per week of any activity, including moderate activity such as walking

When comparing purchases of antidepressants, those living alone had purchased antidepressants more often than those who did not live alone (9.1% versus 5.7%) at the baseline examination. Likewise, during the follow-up period, 25.4% of persons living alone versus 15.8% of others had purchased antidepressants in any year between 2002 and 2008. As shown in Table 3 living alone was associated with a 1.61-fold higher (CI = 1.12-2.25) purchase rate of antidepressants at baseline and 1.81-fold higher (CI = 1.46-2.23) rate during the 7 following years compared with people who did not live alone. When the analyses were restricted to participants who started antidepressant use after the baseline examination (incident use), the association remained similar (OR = 1.71, CI = 1.32-2.21).

**Table 3:** Odds ratios (OR) for use of antidepressants in participants living alone compared to participants not living alone

	Use of antidepressant medication					
	
	At baseline		During 7-year follow-up		**Incident 7-year use**^**a**^	
	
Predictor: living alone (ref. = not alone)	OR (95% CI)	% reduction	OR (95% CI)	% reduction	OR (95% CI)	% reduction
Adjustment in addition to age and gender:						
1. None	1.61 (1.15-2.25)	0	1.81 (1.46-2.23)	0	1.71 (1.32-2.21)	0
2. Psychosocial factors^b^	1.43 (1.03-1.99)	30	1.71 (1.38-2.11)	12	1.67 (1.29-2.16)	6
3. Sociodemographic factors^c^	1.48 (1.04-2.09)	18	1.64 (1.32-2.05)	21	1.57 (1.21-2.05)	20
4. Health behaviors^d^	1.58 (1.25-2.21)	5	1.74 (1.41-2.14)	9	1.64 (1.26-2.12)	10
5. All of the above	1.33 (0.93-1.89)	46	1.55 (1.23-1.94)	32	1.53 (1.16-2.00)	25

Data: The Health 2000 Study, n = 3471

^a^New users during the follow-up with no purchases at the baseline (in 2000 or 2001)

^b^Low social support at the workplace, low social support in private life, poor job climate, hostile personality

^c^Low educational level, low occupational grade, low income, unemployement, urbanicity, living at rent, housing disadvantages

^d^Regular smoking, alcohol heavy use (> 20 g women, > 40 g men), sedentary lifestyle, obesity (BMI > 30)

Sociodemographic factors attenuated these associations by 18%, 21%, and 20%. As shown in Table 3 the ORs adjusted for sociodemographic factors were 1.48 (CI = 1.04-2.09) for baseline antidepressant use, 1.64 (CI = 1.32-2.05) for 7-year use, and 1.57 (CI = 1.21-2.05) for incident antidepressant use. Psychosocial factors attenuated the associations by 30%, 12%, and 6%, with the adjusted ORs being 1.43 (CI = 1.03-1.99) for baseline use, 1.71 (CI = 1.38-2.11) for 7-year use, and 1.67 (CI = 1.29-2.16) for incident antidepressant use. Finally, health behaviors had only a marginal effect on the associations between living arrangements and antidepressant use, with adjusted ORs of 1.58 (CI = 1.25-2.21), 1.74 (CI = 1.41-2.14), and 1.64 (CI = 1.26-2.12), corresponding to a contribution of 5% to 9% owing mainly to heavy alcohol use.

There was no statistical evidence to suggest that gender modifies the association between living arrangements and antidepressant use (*P*-values for gender × living arrangement interactions were 0.618, 0.673, and 0.984). However, the underlying mechanisms could be gender-specific. Thus, we ran the analyses shown for the total cohort in Table 3 stratified by gender to examine whether the contributing factors differed between men and women. Table 4 shows that in men, psychosocial factors had the greatest contributing effect on the association between living arrangements and antidepressant use (attenuations were 31%, 21%, and 15%). In women, sociodemographic factors stood out as the major contributors, showing attenuations of 24%, 27%, and 31%.

**Table 4:** Odds ratios (OR) for use of antidepressants in participants living alone compared to participants not living alone, separately for men and women

	Use of antidepressant medication					
	
	At baseline		During 7-year follow-up		**Incident 7-year use**^**a**^	
	
Predictor: living alone (ref. = not alone)	OR (95% CI)	% reduction	OR (95% CI)	% reduction	OR (95% CI)	% reduction
	**Men (n = 1695)**					

Adjustment in addition to age and gender:						
1. None	1.85 (1.06-3.25)	0	1.72 (1.21-2.44)	0	1.62 (1.09-2.41)	0
2. Psychosocial factors^b^	1.59 (0.89-2.83)	31	1.57 (1.09-2.25)	21	1.53 (1.02-2.29)	15
3. Sociodemographic factors^c^	1.73 (0.96-3.09)	14	1.65 (1.15-2.37)	10	1.62 (1.08-2.43)	0
4. Health behaviors^d^	1.81 (1.03-3.18)	5	1.64 (1.15-2.34)	11	1.56 (1.04-2.33)	10
5. All of the above	1.56 (0.86-2.85)	34	1.51 (1.04-2.20)	29	1.52 (1.00-2.31)	16
	**Women (n = 1776)**					

Adjustment in addition to age and gender:						
1. None	1.54 (1.00-2-39)	0	1.89 (1.40-2.54)	0	1.74 (1.24-2.45)	0
2. Psychosocial factors^b^	1.43 (0.91-2.22)	20	1.81 (1.34-2.45)	9	1.71 (1.21-2.42)	4
3. Sociodemographic factors^c^	1.41 (0.89-2.22)	24	1.65 (1.21-2.25)	27	1.51 (1.05-2.15)	31
4. Health behaviors^d^	1.50 (0.97-2.32)	7	1.83 (1.36-2.46)	7	1.70 (1.20-2.39)	5
5. All of the above	1.29 (0.80-2.06)	46	1.58 (1.15-2.17)	35	1.49 (1.03-2.14)	34

Data: The Health 2000 Study, n = 3471

^a^New users during the follow-up with no purchases at the baseline (in 2000 or 2001)

^b^Low social support at the workplace, low social support in private life, poor job climate, hostile personality

^c^Low educational level, low occupational grade, low income, unemployement, urbanicity, living at rent, housing disadvantages

^d^Regular smoking, alcohol heavy use (> 20 g women, > 40 g men), sedentary lifestyle, obesity (BMI > 30)

Finally, we conducted a sensitivity analysis to examine whether the associations were dependent of the length of the follow-up period. We repeated the analysis of living arrangements and antidepressant use for a 2-year instead of a 7-year follow-up period. The association was directionally similar but slightly weaker than in the main analysis (Additional file 2: Annex Table 2). The contributing factors were similar in magnitude to those in the main analysis, with sociodemographic factors having the greatest contribution and health behaviors the lowest contribution to the greater antidepressant use of participants living alone.

## Discussion

This study suggested that living alone is associated with the prospective use of antidepressant medication in a nationally representative Finnish working-age sample. Those who lived alone had an 80% higher risk of initiating antidepressant use during the 7-year follow-up compared with participants who did not live alone. Socioeconomic adversity explained part of this relationship, especially in women. Psychosocial factors, including a lack of social support, were important explanatory factors in men. Health behaviors had only a marginal contribution to the association between living alone and antidepressant use among men and women, with the exception of heavy alcohol use in men. This is in agreement with a previous register-based study showing a strong link between living alone and alcohol-related mortality [36]. All the factors included in this study explained 46% of the associations, thus leaving the majority of the association between living alone and antidepressant use unexplained.

Our findings are in line with previous reports suggesting that single people suffer from ill health due to material and socioeconomic disadvantages [19,21]. In our study, urban living, poor housing conditions, and rental living contributed to the association between living alone and antidepressant use. Their effect was more pronounced in women than men. A systematic review demonstrated that the very same environmental factors - housing quality, housing tenure, and urban living - had the strongest mental health effects [37]. The direction of causality in our study may go either way: poor living conditions may cause depression, but they may also be a consequence of earlier mental health problems.

A lack of social support, a poor job climate, and a hostile personality were among the psychosocial factors that were associated with living alone and antidepressant use. A lack of supportive social contacts at work and in private life explained part of the association between living alone and antidepressant use in men. Previously, social problems at work and in private life have been associated with antidepressant consumption [24,27]. The concept of "social capital" may offer a theoretical framework for interpreting these findings. Living alone may be associated with less social capital [13,19-22], which, in turn, is a risk for mental health [23-25]. As hostility is a rather enduring personality characteristic associated with irritability, lack of trust, and negative social interactions [38], reverse causation may also play a role. Hostile personalities may be more likely to end up being without a partner due to unwillingness or a lack of skills to form warm and close social relations.

Our data were limited firstly due to the systematic drop-out, causing healthier and economically better-off individuals to be slightly over-represented. This may have restricted the variance in both the predictor variable (living arrangement) and in the outcome variable (antidepressant use), thus underestimating their effects. Second, we were unable to examine whether changes in living arrangements had an effect on antidepressant use. As we had no data on living status on follow-up, some misclassification was possible due to changes in living status after baseline, that is, someone who was originally living alone may have been co-habiting at the time of purchase of antidepressants. Although the main findings were replicated in sensitivity analyses over a shorter time-span, when a change in living status was less likely, this question remains to be examined in further studies. Moreover, we had no data on antidepressant use before the baseline measurement, and thus were unable to examine reverse causation, that is, whether people with prior depression are likely to drift into living alone. Although we showed that living arrangements were associated with starting to use antidepressants (incident use), inferences on whether living alone *causes *mental health problems are not possible based on this observational data set. Third, different reasons for living alone may be differently related to mental health, but we were unable to compare individuals who unwillingly lived alone with those living alone through choice. Neither were we able to measure the effect of different household compositions on mental health, such as the presence of children, a spouse, or elderly relatives. A large prospective study has shown that under certain circumstances, living with other people may be more stressful than living alone [14]. Future studies should examine the possible benefits of living alone, and the advantages and disadvantages of having other persons with different statuses in the household.

Furthermore, antidepressant use as the outcome variable may reflect help-seeking behavior or differences in access to health care. People using antidepressants have depression that has been diagnosed by a health care professional, and people with undiagnosed depression were not therefore identified. Moreover, people typically seek help if the disease is severe enough to cause functional disability [39], suggesting that participants with mild depressive symptoms may have been undetected. This may have resulted in underestimation of the number of depressed people and underestimation of the effects. Finally, some antidepressants, particularly tricyclic medication, are commonly used for non-psychiatric indications such as pain or sleeping problems [12,40]. However, it is unlikely that the indication of antidepressant use varies according to living arrangements [12].

The strengths of this study were that common rater variance was excluded because the sources of information on the outcome (national register-based data) and contributing factors (self-reported data) were independent of each other. Because all purchases of antidepressants are recorded in the national registers in Finland, we were able to track 100% of participants who had purchased antidepressant medication at least once during the study years (excluding medications used during hospitalization). We included a rather extensive set of well-established risk factors for mental health problems. To our knowledge, this is the first study to examine both private life and work life factors as contributors to the association between living arrangements and mental health outcomes within the same sample.

## Conclusion

This study focused on a working-age population and its antidepressant use over almost a decade. Persons living alone faced several types of psychosocial and material disadvantage. We found evidence to suggest that the explanatory factors may be gender specific, with a lack of social support playing a greater role in men and socioeconomic disadvantages in women. These finding suggest that improving the quality of social relations and material circumstances should be important targets in mental health promotion. Further research is needed, as over half of the association between living alone and antidepressant use remained unexplained by the variables included in this study. Further insights may arise, for example, from assessments that cover social capital (that is, feelings of alienation and a lack of trust in society), critical life events and their accumulation over time, and childhood circumstances giving rise to psychological vulnerabilities in adulthood.

## Competing interests

The authors declare that they have no competing interests.

## Authors' contributions

L P-R was the principal investigator, conducted the data analyses, wrote the first draft, and will act as guarantor for the paper. MK, KA, KJ, ME, HR, SP, SK, JL and MV substantially contributed to conception and design of the study, interpretation of data, drafting the article critically for important intellectual content, and approved of the final version. All authors read and approved the final manuscript.

## Pre-publication history

The pre-publication history for this paper can be accessed here:

http://www.biomedcentral.com/1471-2458/12/236/prepub

## Supplementary Material

Additional file 1 **Prevalence and incidence of antidepressant use according to follow-up year**. The Health 2000 Study, n = 3471. Click here for file

Additional file 2 **Odds ratios (OR) for use of antidepressants during a 2-year follow-up period in participants living alone compared to participants not living alone**. Data: The Health 2000 Study, n = 3471. Click here for file
