# Supplementary material for: Living alone and antidepressant medication use: a prospective study in a working-age population
Source: BMC Public Health. 2012 Mar 23;12:236. doi: 10.1186/1471-2458-12-236 (PMC3338384; doi:10.1186/1471-2458-12-236)
Supplement: Additional file 1 — Prevalence and incidence of antidepressant use according to follow-up year. The Health 2000 Study, n = 3471. [file 1471-2458-12-236-S1.PDF]

Annex table 1 (on-line supplement)

Prevalence and incidence of antidepressant use according to follow-up year. The Health 2000 Study, n=3471.

Follow-up year	Prevalence n (%)	New users[a] n (%)
2002	186 (5.4)	65 (1.9)
2003	212 (6.1)	69 (2.0)
2004	240 (6.9)	58 (1.7)
2005	238 (6.9)	49 (1.4)
2006	254 (7.3)	53 (1.5)
2007	168 (7.7)	57 (1.6)
2008	309 (8.9)	69 (2.0)

[a]Indicates first-time users who had no previous purchases of antidepressants at any year.
