# Supplementary material for: Living alone and antidepressant medication use: a prospective study in a working-age population
Source: BMC Public Health. 2012 Mar 23;12:236. doi: 10.1186/1471-2458-12-236 (PMC3338384; doi:10.1186/1471-2458-12-236)
Supplement: Additional file 2 — Odds ratios (OR) for use of antidepressants during a 2-year follow-up period in participants living alone compared to participants not living alone. Data: The Health 2000 Study, n = 3471. [file 1471-2458-12-236-S2.PDF]

Annex table 2 Odds ratios (OR) for use of antidepressants during a 2-year follow-up period in participants living alone compared to participants not living alone. Data: The Health 2000 Study, n = 3471

	Use of antidepressant medication					
	At baseline		During 2-year follow-up		Incident 2-year use[a]	
Predictor: living alone (reference group=not alone	OR (95% CI)	% reduction	OR (95% CI)	% reduction	OR (95% CI)	% reduction
Adjustment in addition to age and gender:						
1.None	1.61 (1.15-2.25)	0	1.41 (1.05-1.89)	0	1.19 (0.80-1.76)	0
2.Psychosocial factors[b]	1.43 (1.03-1.99)	30	1.29 (0.96-1.73)	29	1.14 (0.77-1.70)	26
3.Sociodemographic factors[c]	1.48 (1.04-2.09)	21	1.25 (0.93-1.69)	39	1.05 (0.70-1.57)	74
4.Health behaviors[d]	1.58 (1.25-2.21)	5	1.35 (1.00-1.81)	15	1.13 (0.76- 1.68)	32
5.All of the above	1.33 (0.93-1.89)	46	1.13 (0.83-1.54)	68	0.99 (0.66-1.50)	na[e]

[a] New users during the follow-up with no purchases at the baseline (in 2000 or 2001).

[b] Low social support at the workplace, low social support in private life, poor job climate, hostile personality.

[c] Low educational level, low occupational grade, low income, unemployement, urbanicity, living at rent, housing disadvantages.

[d] Regular smoking, alcohol heavy use (>20g women, >40g men), sedentary lifestyle, obesity (BMI >30)
